# Improving UV Resistance of Aramid Fibers by Simultaneously Synthesizing TiO_2_ on Their Surfaces and in the Interfaces Between Fibrils/Microfibrils Using Supercritical Carbon Dioxide

**DOI:** 10.3390/polym12010147

**Published:** 2020-01-07

**Authors:** Hui Sun, Haijuan Kong, Haiquan Ding, Qian Xu, Juan Zeng, Feiyan Jiang, Muhuo Yu, Youfeng Zhang

**Affiliations:** 1 School of Materials Engineering, Shanghai University of Engineering Science, Shanghai 201600, China; sunhui1196@163.com (H.S.); dinghaiquan0502171@126.com (H.D.); answerxu227@126.com (Q.X.); zj2470529861@163.com (J.Z.); jiangfeiyan1996@163.com (F.J.); liannishang@126.com (Y.Z.); 2 Shanghai Collaborative Innovation Center of Laser Advanced Manufacturing Technology, Shanghai 201600, China; 3 State Key Laboratory for Modification of Chemical Fibers and Polymer Materials, College of Material Science and Engineering, Donghua University, Shanghai 200051, China; yumuhuo@dhu.edu.cn

**Keywords:** aramid III fiber, TiO_2_, supercritical carbon dioxide, UV Resistance

## Abstract

Aramid fibers with low density and high strength, modulus, and thermal resistance are widely used in applications such as bulletproof vests and cables. However, owing to their chemical structure, they are sensitive to ultraviolet light, which degrades the fibers’ useful mechanical properties. In this study, titanium dioxide (TiO_2_) nanoparticles were synthesized both on the aramid III fiber surface and in the interfacial space between the fibrils/microfibrils in supercritical carbon dioxide (scCO_2_) to improve the UV resistance of aramid fibers. The effects of scCO_2_ treatment pressure on the TiO_2_ structure, morphology, surface composition, thermal stability, photostability, and mechanical properties were investigated using Fourier transform infrared spectroscopy, X-ray diffraction, scanning electron microscopy, X-ray photoelectron spectroscopy, thermogravimetric analysis, ultraviolet–visible spectroscopy, and single-fiber test. The results show that amorphous TiO_2_ formed on the fiber surface and the interface between fibrils/microfibrils, and decreased the photodegradation rate of the aramid III fiber. Moreover, this modification can also improve the tensile strength via treatment at low temperature and without the use of a solvent. The simple synthesis process in scCO_2_, which is scalable, is used for mild modifications with a green solvent, providing a promising technique for synthesizing metal dioxide on polymers.

## 1. Introduction

Aramid III fiber (AF-III) has high strength, i.e., up to about 6 GPa, a modulus of up to 230 GPa, and a density of 1.44 g/cm^3^; it is widely used in many applications, such as bulletproof vests, firefighting gear components, ropes and cables, and fiber-reinforced composite applications. Therefore, it is an indispensable strategic material for national security, construction, and scientific and technological progress [1,2]. Unfortunately, it is sensitive to ultraviolet (UV) radiation, which leads to serious degradation. It has been found that exposing the fiber to UV radiation results in significant damage, such as mass loss, deterioration of mechanical properties, color changes, and changes in the morphology and structure, that in turn lead to a reduction in performance, causing premature failure and limiting the material’s potential for outdoor use [3,4,5,6]. Therefore, we need to improve the UV resistance performance of aramid III fiber, and extend its outdoor service life. Owing to the difficulty in dealing with high-performance fibers, UV stabilizers, be they organic or inorganic (TiO_2_, ZnO), cannot be loaded during spinning due to the harsh conditions of the spinning process. The high viscosity of the fiber-spinning solution makes it difficult to disperse and oxidize UV stabilizers. Furthermore, because of the highly oriented packed crystalline structure, it is difficult to incorporate UV stabilizers into the fibers. This is why protecting high-performance fibers from UV is a serious challenge for material scientists and engineers.

To overcome these undesired characteristics, many researchers have sought to improve photostability by loading UV stabilizers. For example, a coating containing a UV stabilizer was applied, or protective polymer layers that are stable to UV radiation were sheathed [7,8,9,10,11,12,13,14]. Nanoscale TiO_2_ effectively protects the fibers from photodegradation by reflecting or scattering UV rays [10,11,15]. Lee used the SiO_2_/TiO_2_ sol-gel method to improve the photodegradation resistance of the fiber, but the adhesion of the surface of the fiber was poor [16]. The photodegradation ability of aramid fibers was improved by a nano-TiO_2_ and Al^3+^ doped TiO_2_ sol-gel method [17]. Park et al. modified para-aramid fiber by TiO_2_ synthesized by the sol-gel method; the size of the TiO_2_ particles was adjusted by adjusting the ratio of titanium isopropanol to water [18]. However, the TiO_2_ coating prepared by the sol-gel method is brittle and has poor durability. Walsh studied UV degradation on polybenzoxazole (PBO) fiber, and found that the degradation was confined to the fiber surface as UV–Vis radiation primarily represents the hydrolysis of the material near the fiber surface, with the attendant formation of amide linkages [19,20].

The aforementioned method to improve the photostability mainly focuses on protecting the surface, but the fiber structure was not specified, which is also important. As we know, the main chain of aramid fibers contain C=C bonds, which can easily absorb UV energy and form the free radical ROO with oxygen in air [6,21,22]. Furthermore, in semicrystalline polymer materials, photodegradation usually occurs in the amorphous state or in the interspace between fibrils/microfibrils, because oxygen atoms easily penetrate these regions, but have difficulty penetrating the crystal region [23]. If these spaces were also modified, the photostability would be fundamentally improved.

Supercritical carbon dioxide (scCO_2_) has many desirable attributes, such as low cost, abundance, low toxicity, and being readily accessible under supercritical conditions (critical temperature *T*_c_ = 31.1 °C, critical pressure *P*_c_ = 7.38 MPa, critical density *ρ*_c_ = 0.472 g/mL). ScCO_2_ has recently gained more popularity in research and industry as an environmentally-friendly solvent and blowing agent in a wide range of applications, including polymerization, polymer purification and fractionation, coating applications, and powder formation [24,25,26,27,28,29,30,31,32,33,34]. L.Z. reported a method to synthesize nano-SiO_2_ on the surface of AF to improve its UV resistance in scCO_2_. But that research mainly focused on surface protection, and did not involve the improvement of the interspace between fibrils/microfibrils of aramid fibers, where light aging also occurs [35]. In our earlier article, we reported a new method for improving the mechanical properties of a single aramid filament by forming a crosslinking network structure in the interface between fibrils/microfibrils of a *p*-phenylene terephthalamide (PPTA) fiber with the aid of scCO_2_, which enhanced the interface between the fibrils/microfibrils and improved the tensile strength and modulus of the fiber [35,36,37,38]. 

In this study, TiO_2_ nanoparticles were synthesized both on the aramid III fiber surface and in the interfacial space between the fibrils/microfibrils. The titanate precursor was first dissolved in scCO_2_, infiltrated into the loosened fibril/microfibrils, and spread on the surface. Then, ethanol was added to cause loaded precursor–fiber hydrolysis in scCO_2_. Nano-TiO_2_ synthesized in situ on the fiber and in the microfibril was obtained. The effects of the pressure of scCO_2_ treatment on the chemical structure, surface morphology, and mechanical properties with UV exposure over time were studied. 

## 2. Materials and Methods

### 2.1. Materials

Aramid III fibers (linear density of 120 tex) were supplied by Zhonglan Chenguang Chemical Research Institute Co., Ltd. (Chengdu, China). Carbon dioxide (purity: +99.99%) was purchased from Shanghai Chenggong Gases Co., Ltd. (Shanghai, China). Tetrabutyl titanate (TBT, purity: 99%) was purchased from Sinopharm Chemical Reagent Co., Ltd. (Shanghai, China). Acetone (purity: +99.5%) was bought from Shanghai Yunli Trading Co., Ltd. (Shanghai, China). Anhydrous ethanol (purity: +99.7%) was bought from Changshu Yeung Yuen Chemical Co., Ltd. (Changshu, China). Acetic acid (purity: +99.7%) was bought from Kunshan Jingke Microelectronics Material Co., Ltd. (Changshu, China). These chemicals were used without further purification.

### 2.2. Synthesis of TiO_2_ in scCO_2_

**Figure 1:**
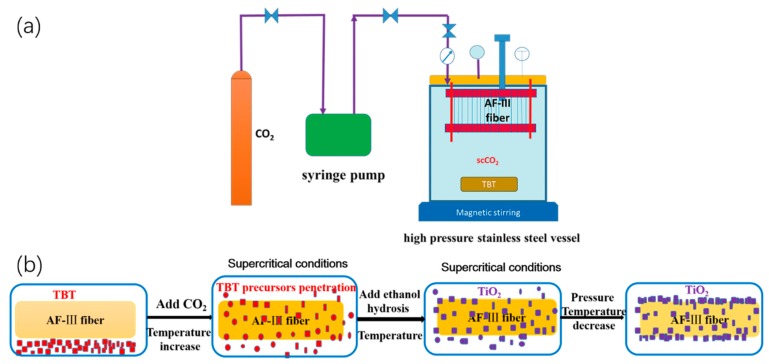
The scheme of the device and TiO_2_ synthesized process: the scheme of the scCO_2_ treatment device (**a**), the process of synthesizing TiO_2_ for AF-III in scCO_2_ (**b**).

The synthesis process was carried out in a 500-mL stainless-steel high-pressure reactor with a syringe pump to pump CO_2_. The scCO_2_ treatment device is shown in Figure 1a. The process comprises two steps. The first is to deposit the TBT on the fiber surface and impregnate it into the interior of the fibers. After being cleaned with acetone, the AF-III fiber was rolled on a stainless-steel framework and placed in the reactor. An appropriate amount of TBT precursors was first placed on a glass filter at the bottom of the reactor. The reactor was pressurized with CO_2_ up to the desired temperature and pressure under stirring. After 2 h, the system was depressurized nearly instantaneously to atmospheric pressure. With the help of the swelling and dissolution of scCO_2_, the TBT was dissolved and impregnated into the fiber; the obtained fiber was called TBT-fiber. 

The second process comprises hydrolysis in scCO_2_ with 30 mL ethanol, 0.1 mL acetic acid, and 3 mL distilled water to obtain TiO_2_-modified aramid fiber. Ethanol and acetic acid were mixed and put in a glass beaker at the bottom of the reactor, and CO_2_ was pumped again to the former pressure. Once the hydrolysis of the TBT deposited on the surface of the fiber or impregnated into the fibers had occurred, TiO_2_ was formed in the interface between the fibrils/microfibrils region in the fiber and on the fiber surface. 

A schematic illustration of a two-step process is shown in Figure 1b. The products were washed with ethanol, acetone, and distilled water (cleaned more than three times). Then, the products were dried in the oven at 100 °C for 2 h. Both steps were carried out in scCO_2_, concluding the penetration and hydrolysis of TBT precursors.

### 2.3. Characterizations

The surface functional groups were studied using Fourier transform infrared spectroscopy (FTIR) (Nicolet 6700, Thermo Fisher Company, New York, NY, USA) in the spectral region of 4000–400 cm^−1^ with a resolution of 2 cm^−1^ at 32 scans. The samples were powdered, mixed with KBr, and processed into pellets. The surface morphology and fracture morphology of the modified aramid III fibers were observed using scanning electron microscopy (SEM) (S-4800, Tokyo, Japan). Before SEM analysis, all samples were coated with a thin conductive layer of gold via vapor deposition to minimize the charge. X-ray diffraction (XRD, Rigaku D/MAX-2550, Tokyo, Japan) was used to determine the crystalline phase of AF-III. The measurement was conducted with a Cu Kα radiation source (λ = 1.5406 Å) with 40 kV and 200 mA), followed by a scanning range of 5.0°–90.0° at a speed of 20 °/min. The element composition of AF-III was analyzed using X-ray photoelectron spectroscopy (XPS, ESCALAB 250Xielectron spectrometer, Thermo Fisher Scientific, New York, NY, USA) with a monochromatic Al Kα source. The pressure in the analysis chamber was controlled at 5 × 10^−8^ Pa. The deconvolution curve fitting of the C1s and Ti2p peaks for fibers was performed using Peakfit software. A UV–visible (UV-Vis) spectrophotometer (UV3600, Shimadzu, Japan) equipped with an integrating sphere was used to measure the absorbance of the fibers in the wavelength range of 200–450 nm. The AF-III fibers were cut into powders. Then, the powder of the fiber was pressed into a thin layer at the center of the sphere and the measurements were performed. A thermogravimetric analyzer (TGA4000, Perkin Elmer, Waltham, MA, USA) was used to measure the thermal stability of the fibers. Temperature ramp measurements were conducted in an inert atmosphere of N2 from 30 to 850 °C at 20 °C·min^–1^. The mechanical properties of the single fibers were carried out using a tensile-strength tester (XQ-1A, Shanghai New Fiber Instrument Co., Ltd., Shanghai, China) with a head speed of 10 mm/min at a gauge length of 10 mm. The average values of the tenacity and modulus were calculated from at least 15 samples. The linear density of the fiber was measured using an XD-1 vibrating fiber-fineness instrument (Shanghai New Fiber Instrument Co., Ltd., Shanghai, China). The unit conversion of Pa and CN/dtex was based on Equation (1).

(1) $$\sigma =9.53\times 10^{7}\times \varrho \times \frac{\mathrm{CN}}{\mathrm{dtex}}\left(\mathrm{Pa}\right)$$

where σ is the tensile strength of Pa unit, *ρ* is the density of the fiber as 1.44 g/cm^3^, dtex is the average linear density for 10,000 meters of fiber. Fibers were exposed to UV radiation using a weathering test machine with a UV lamp (UVB 280-315 nm, 40 W, lamp length of 1220 mm, Dongguan Instrument Co. Ltd., Guangzhou, China) to study the effect of UV aging over time on the mechanical properties of the fibers. The distance between the fiber sample and the UV lamp was 20 cm. The fibers were placed in parallel rows in the sample tray which was exposed to 40 W/m^2^ and held at a relative humidity of 60% for 168 h, according to the Chinese Standard GB/T 14522-93.

## 3. Results and Discussion

### 3.1. Influence of the Treatment on the Structures of AF-III Fibers

#### 3.1.1. Crystalline Structure

**Figure 2:**
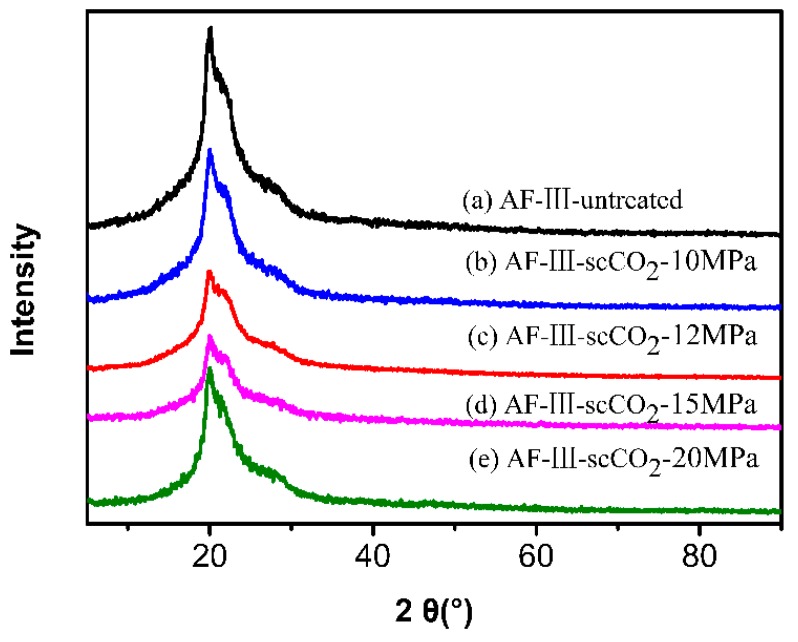
XRD patterns of untreated fiber (**a**) and nano-TiO_2_ modified fibers prepared in scCO_2_ under pressures of 10 MPa (**b**), 12 MPa (**c**), 15 MPa (**d**), and 20 MPa (**e**).

XRD was applied to detect the TiO_2_ crystal phase. As shown in Figure 2, there are two diffraction peaks at 20.1° and 21.9° due to the (110) and (200) planes, respectively, for the untreated AF, corresponding to the crystalline domains of aramid fibers [39]. The crystalline phases of TiO_2_ were not found in the XRD curves of the modified fibers, which demonstrated that the TiO_2_ formed on the fibers was amorphous. At the same time, the more TiO_2_ particles contained on the fiber surface and in the interfacial space between the fibrils/microfibrils, the lower the crystallinity of the fiber; this is related to the change of pressure. With the increase of pressure, the content of TiO_2_ prepared on the surface and in the interfacial space increased gradually. As shown in Figure 2, the crystallinity of the sample prepared under 15 MPa was the lowest, which indicated that the particles of TiO_2_ prepared under 15 MPa were most abundant. However, when the pressure is too high, i.e., up to 20 MPa, the content of TBT is also too high. TiO_2_ particles aggregate under the influence of intermolecular force, forming uneven, large particles, which leads to shedding on the surface of the fiber. Due to its excellent ability of absorbing and shielding from ultraviolet light, TiO_2_ particles were shown to play an important role in protecting aramid fiber from ultraviolet aging [40].

#### 3.1.2. FTIR Analysis

**Figure 3:**
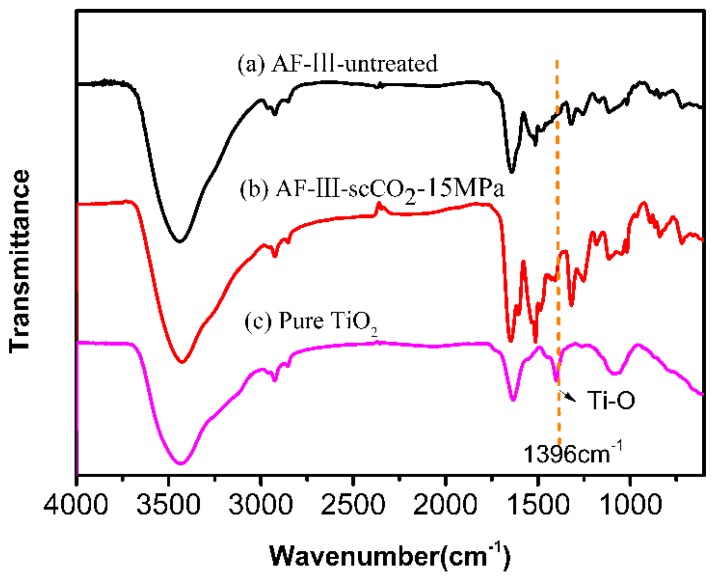
FTIR curves of untreated fiber (**a**) and nano-TiO_2_ modified fiber prepared in scCO_2_ under a pressure of 15 MPa (**b**) and the pure TiO_2_ (**c**).

The FTIR spectra of untreated and treated AF-III fibers are presented in Figure 3. The spectrum of all fibers showed three peaks at 3300 cm^−1^ (–NH stretching), 1640 cm^−1^ (–C=O– group), and 1540 cm^−1^ (–NH group of bending). After modification by TiO_2_ in scCO_2_, the peak intensity at 1640 cm^−1^ became stronger than that of the untreated fiber. Compared with pure TiO_2_, we know that stretching vibrations of Ti–O occur at 1396 cm^−1^. It was shown that TiO_2_ was successfully synthesized on the fiber surface.

#### 3.1.3. Surface and Interior Morphology of the Fibril/Microfibril

**Figure 4:**
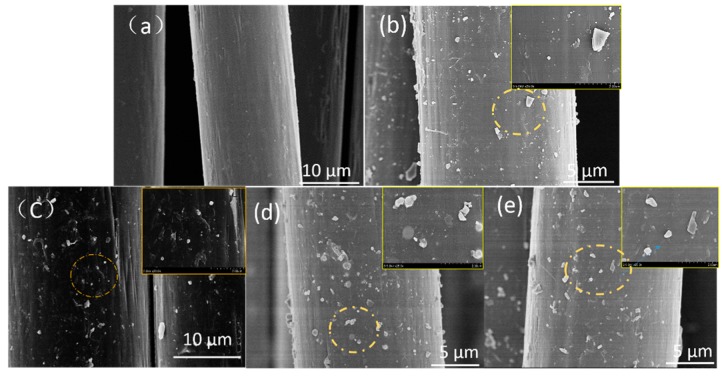
SEM images of untreated fiber (**a**) and nano-TiO_2_ modified fibers prepared in scCO_2_ under pressures of 10 MPa (**b**), 12 MPa (**c**), 15 MPa (**d**), and 20 MPa (**e**).

The surface morphology of aramid fibers was studied using SEM. In Figure 4, the SEM images of the untreated and modified fibers are shown under various pressures in scCO_2_. It can be seen that the surface of the untreated fibers (Figure 4a) is smooth and clean. Compared to untreated aramid fiber, the surface of the modified aramid fiber is rough and has many obvious nanoparticles on the surface, as shown in Figure 4b–e. Combining the results of the XPS and FTIR, we can conclude that these nanoparticles are TiO_2_. When the treatment is at a pressure of 10 MPa, less TiO_2_ forms on the surface, and the distribution and size are uneven. The results showed that, when treated at a pressure of 15 MPa, TiO_2_ is well synthesized and packed on the surface. At the same time, the TiO_2_ particles formed on the fiber surface were most abundant, and agglomeration was reduced. However, when the treatment pressure increased to 20 MPa, fewer particles attached to the surface and the size of the TiO_2_ particles was largely due to the agglomeration. The main cause of this phenomenon is related to the solubility of scCO_2_; the higher the pressure, the denser the distribution of the molecules. As the pressure increases, the CO_2_ concentration also increases, leading to an increase in the dissolution amounts and diffusion rate of the TBT in scCO_2_. But when the pressure is too high, the TBT content will also be too high. When the CO_2_ is released, the rapid relative motion between the particles and the gas causes the TiO_2_ particles to agglomerate into large particles under the intermolecular force, and adhere unevenly to the surface of the fiber [41]. Since the large particles easily fall off the surface of AF-III; under external force, the content of nano-TiO_2_ particles under a pressure of 20 MPa was less than that at 15 MPa. The above results are consistent with those of XPS.

**Figure 5:**
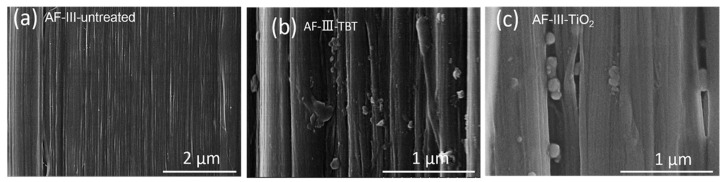
SEM images of microfibril/fibril structure of AF-III fiber after longitudinal tearing: (**a**) untreated, (**b**) AF-III-TBT impregnated in scCO_2_, (**c**) AF-III-TiO_2_ in scCO_2_.

To investigate the formation inside the fibers, the fiber was longitudinally torn along the fiber direction. As shown in Figure 5, there are many nano-TiO_2_ particles attached to the supercritical-treated aramid fiber compared to the untreated aramid fiber. Figure 5b shows the internal structure of aramid fibers with precursor, and Figure 5c shows the morphology after hydrolysis. As shown in Figure 5b, the particles impregnated the interspace of the fibrils/microfibrils. In Figure 5c, the particles become smaller and had a regular morphology with the TBT fiber after hydrolysis. The results show that nano-TiO_2_ particles had formed inside the aramid fiber.

#### 3.1.4. XPS Analysis

**Figure 6:**
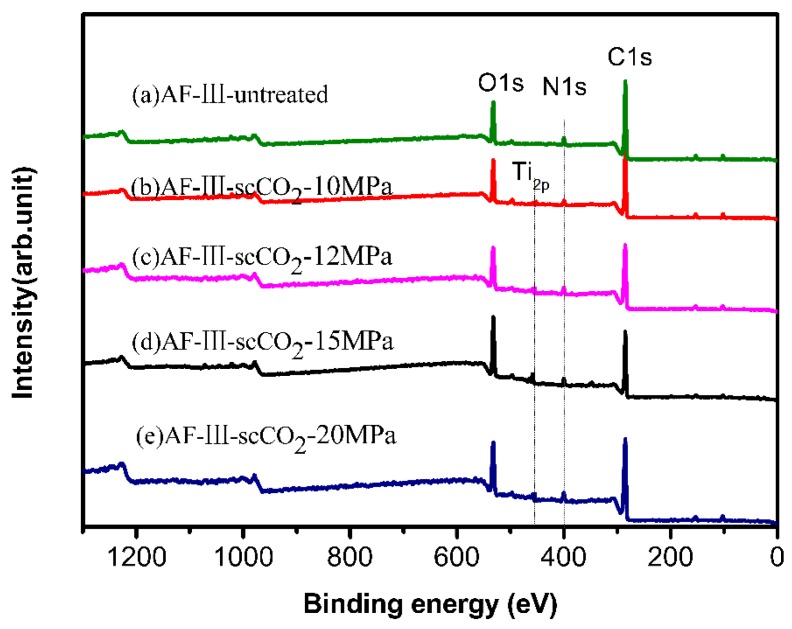
XPS spectrum of untreated fiber (**a**) and nano-TiO_2_ modified fibers prepared in scCO_2_ under pressures of 10 MPa (**b**), 12 MPa (**c**), 15 MPa (**d**), and 20 MPa (**e**).

**Figure 7:**
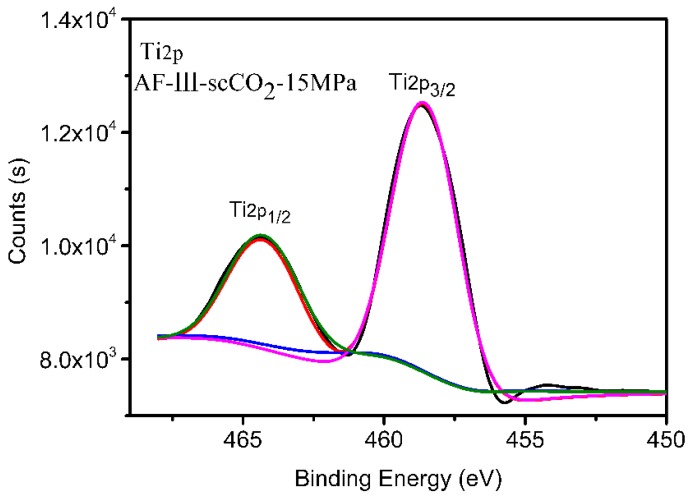
The XPS spectrum of Ti_2p_ of treated fibers in scCO_2_ under a pressure of 15 MPa.

To investigate the influence of supercritical pressure on the surface of aramid fibers, the surface composition of the fibers under different pressures was determined using XPS. It was shown that the oxygen contents of the surfaces of aramid fibers prepared under different pressures were higher than those of untreated fibers. As shown in Figure 6, C_1s_, O_1s_, and N_1s_ peaks appear at 284.7, 531.8, and 400.3 eV, respectively, which represent the presence of C, N, and O in the aramid. Compared with the untreated aramid fiber, the modified aramid fiber showed a new peak of titanium atoms at 453.4 eV, which means that TiO_2_ was successfully prepared on the surface. The results of the elemental content changes are shown in Table 1. Compared with the untreated AF-III fibers, the contents of C and N in the modified aramid decreased, while those of O and Ti increased. When the treatment pressure was 15 MPa, the O and Ti contents were the greatest, and the ratio of O:C increased from 0.2269 to 0.3711; also the content of elemental Ti was the highest, i.e., up to 2.66%. The results were consistent with the results shown in Figure 4, which indicated that the content of TiO_2_ prepared at 15 MPa was the highest. This means that TiO_2_ can be synthesized as much as possible on the surface of AF-III when the appropriate supercritical pressure is reached. Figure 7 depicts the XPS spectrum of Ti_2p_ in the treated fibers in scCO_2_ under a pressure of 15 MPa. The peaks at 464.5 eV for Ti_2p 1/2_ and at 458.6 eV for Ti_2p 3/2_ can be observed in Figure 7. These are typical binding energies for the Ti^4+^ species, showing good agreement with those of the ordinary TiO_2_ [42,43,44]. These results further confirmed that TiO_2_ was synthesized on the surface of fiber.

#### 3.1.5. Mass of Added TiO_2_ on the Surface of Aramid Fibers

Table 2 shows the TiO_2_ at different masses added under the different treatment pressures. It was found that the mass of the added TiO_2_ increases with increasing fiber mass. The results agree with the observed higher area covered by TiO_2_. However, the weight of added TiO_2_ varied with pressure. When the pressure was 15 MPa, the TiO_2_ added was 2.38%, which was the largest increase. This may be because with increasing pressure, the dissolution and impregnation of TBT in scCO_2_ increased. However, the pressure is too high at 20 MPa, and the amount of added TiO_2_ was not the largest. The reason for this is that TiO_2_ would agglomerate to form large particles at the higher pressure that cannot uniformly coat the fiber surface, and which would easily be removed by washing or further characterization. 

### 3.2. Mechanical Properties of AF-III

**Figure 8:**
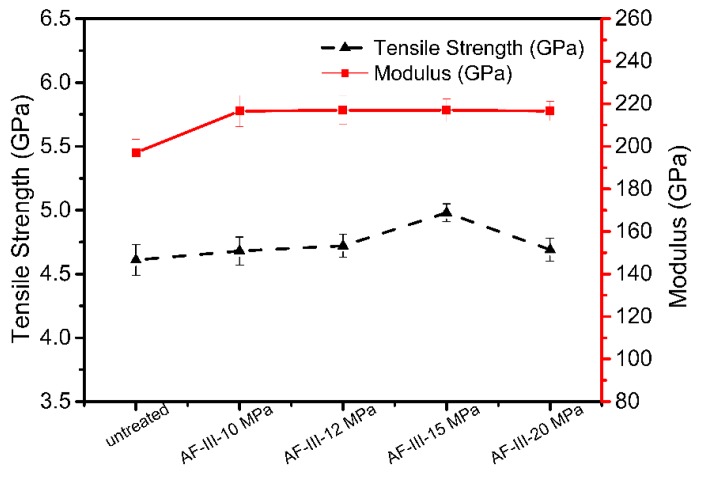
The tensile strength and modulus of the fibers treated in scCO_2_.

The effects of treatment pressure on the mechanical properties of aramid fibers are shown in Figure 8. It was found that both the tensile strength and modulus of AF-III fibers treated at different pressures were higher than those of the untreated fibers, and the strength and modulus of aramid fibers increased with an increase in reaction pressure. When the reaction pressure was 15 MPa, the strength and modulus of the fibers reached the maximum value. When the pressure was 20 MPa, the strength and modulus showed a lesser increase, but it was still higher than that of untreated fiber. With the increase of pressure, the CO_2_ molecular density also increased and more TBT was dissolved in scCO_2_, which infiltrated the fiber and adhered to the fibrillar elements, thus providing a secondary means by which to improve the mechanical properties. Moreover, the tensile modulus clearly improves because the molecular chains tend to arrange themselves with the help of the plasticization of scCO_2_ and tension; however, when the pressure is too high, it may decrease in volume and mobility, so the strength and modulus are not noticeably improved. 

### 3.3. UV-Resistance Analysis

#### 3.3.1. UV-Vis Analysis

**Figure 9:**
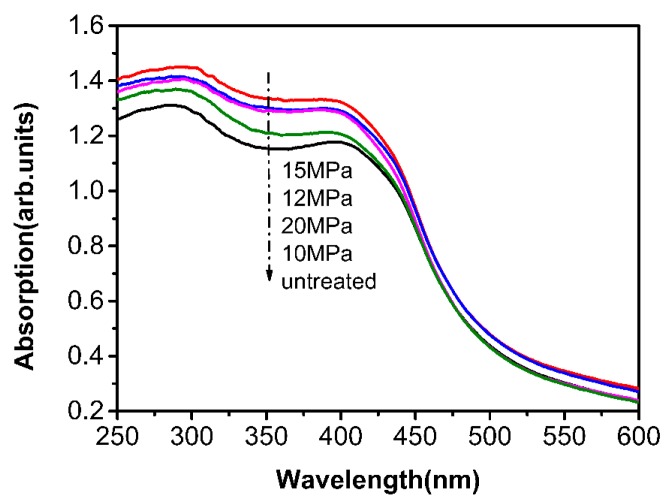
UV-Vis curves of untreated fiber and nano-TiO_2_ modified fibers in scCO_2_.

Figure 9 shows that the UV-Vis spectrum of AF-III is in the range of 250–600 nm, of which 250–400 nm is the UV-absorption region and 400–600 nm is the visible absorption region. Compared to the untreated fibers, treated fibers have significant UV shielding because the nano-TiO_2_ could absorb the UV light such that the anti-UV ability of modified fiber is enhanced. The results demonstrate that the ultraviolet absorption effect at 15 MPa is the best, which might be due to the high levels of loaded TiO_2_. However, the UV absorption of fibers at 12 and 20 MPa is slightly different; this trend is not in agreement with the mass of the loaded TiO_2_. The results showed that the UV-Vis absorption is not only related to the mass of the TiO_2_, but also to the size and distribution of its particles. 

#### 3.3.2. Mechanical Properties

**Figure 10:**
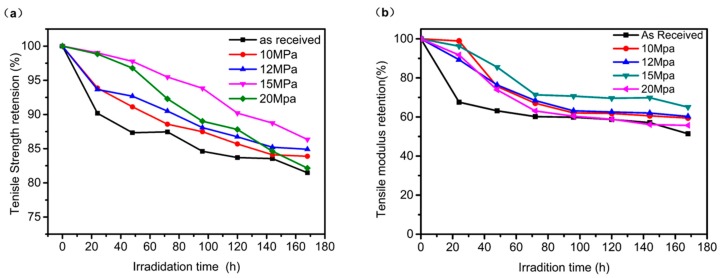
Mechanical properties of untreated fiber (**a**) and nano-TiO_2_ modified fibers (**b**) after UV irradiation.

The mechanical properties of AF-III fibers modified in scCO_2_ under different pressures irradiated by a UV lamp for different times were studied. As shown in Figure 10, there is a clear decreasing trend in the tensile strength and modulus for these fibers, both treated and untreated, with increasing exposure time. With a treatment pressure at 15 MPa, the fibers retained better tensile strength than those at pressures of 10, 12, and 20 MPa. The results are consistent with the UV-Vis spectroscopy results.

The tensile strength and modulus of untreated aramid fibers remained 81.48% and 51.41%, respectively, after 168 h of UV irradiation. The fibers modified with TiO_2_ in scCO_2_ showed less strength and modulus loss than untreated fibers after exposure to UV-Vis radiation, especially at 24 h. The fibers treated at 15 MPa showed 86.34% tensile strength and 65.03% modulus after 168 h, which is a 5%–14% improvement compared to those of untreated AF-III. Furthermore, as Figure 8 shows, the tensile strength and modulus of the modified fiber were improved, so after exposure to UV-Vis, they all showed better mechanical properties. The results showed that an in situ preparation of titanium dioxide on the surface and in the interface between fibrils/microfibrils in scCO_2_ improved not only the tensile strength, but also the UV-Vis resistance.

#### 3.3.3. Surface Compositions 

In order to further study the effect of the TiO_2_ on the aging performance of the aramid fiber, the change of the surface composition before and after the aging of aramid fiber was determined using XPS. 

**Figure 11:**
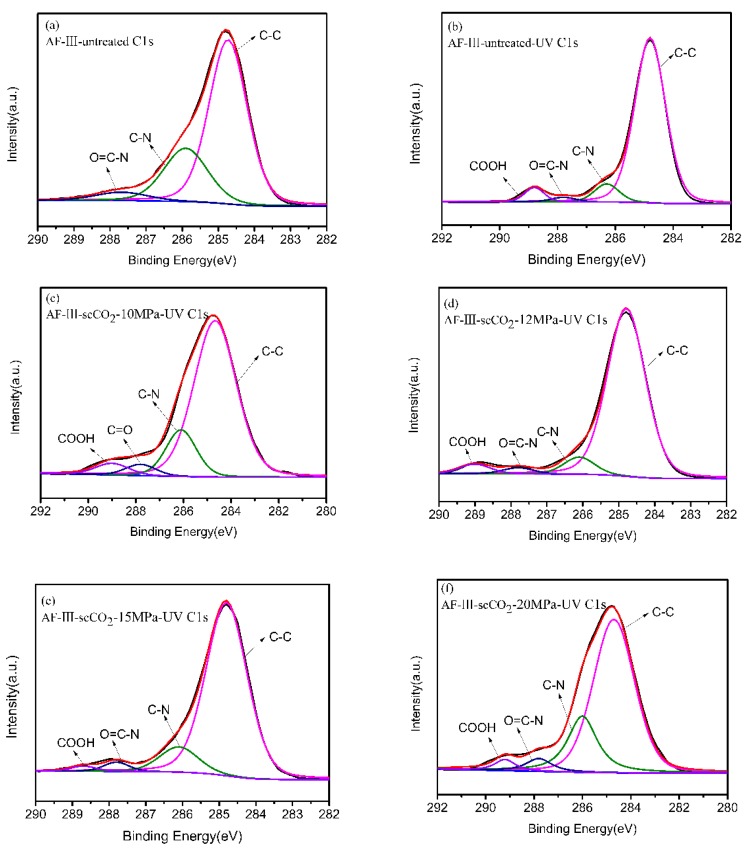
C1s core-level spectra of AF-III fibers and UV irradiated AF-III fibers.

Figure 11 shows the C1s core-level spectra of AF-III fibers and UV irradiated AF-III fibers under different pressures. The components of untreated AF-III fibers (Figure 10a) can be divided into three peaks, i.e., 284.6 eV (C–C), 285.9 eV (C–N) and 287.8 eV (N–C=O). After 168h UV radiation, a new peak associated with the COOH group was observed at a binding energy of 289.8 eV for the irradiated untreated AF-III fibers (Figure 11b), demonstrating that aging absorbs UV energy, leading to the formation of the COOH group [17,45,46]. Compared with the COOH peak of AF-III-untreated-UV fibers, AF-III-scCO_2_-UV fibers (Figure 11c–f) shows relatively insignificant weak peaks. The results show that the nano-TiO_2_ particles formed on the surface of the fiber can shield a large amount of ultraviolet light. Notably, this peak is almost invisible in AF-III-scCO_2_-15MPa-UV fibers (Figure 11e), which indicates that AF-III fibers are more effectively protected by scCO_2_ treatment at 15 MPa.

### 3.4. Thermogravimetric Analysis (TGA)

**Figure 12:**
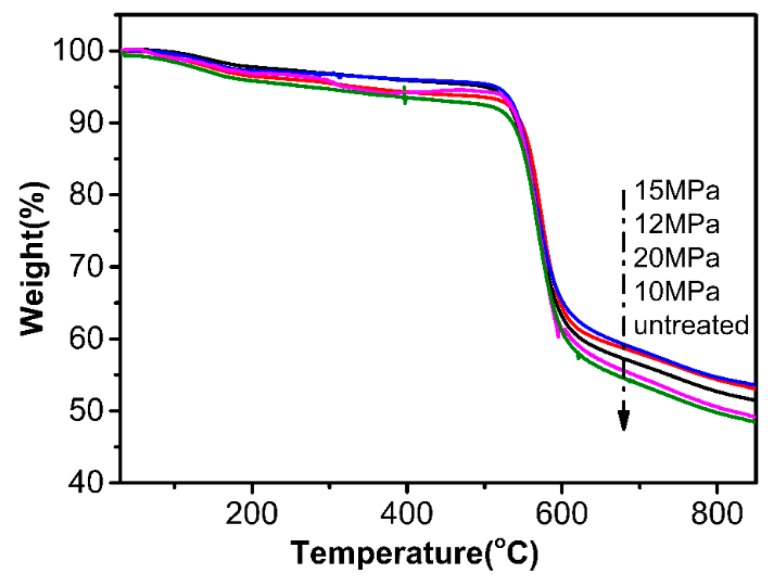
TGA curves of untreated fiber and nano-TiO_2_ modified fibers in scCO_2_.

Figure 12 shows a decrease in the initial weight loss of the fiber below 850 °C. Before 500 °C, fibers treated at 10, 12, 15, and 20 MPa had higher residual masses than the original fibers. The results show that the heat resistance of the treated fiber was improved.

The first interval is the microgravity stage, which is mainly the process of losing the intermolecular bound water. From this range, it can be concluded that there is no significant difference in the content of molecular binding water between the two aramid fibers. The second stage is the thermal decomposition stage (500–600 °C), and the onset of the fiber decomposition is almost the same. The results demonstrate that there is less effect on the stability of the fiber after coating with TiO_2_. 

The third zone (600–850 °C) is the stable stage of carbon formation, in which the fiber is carbonized and the weight loss of the residue is small. Compared to untreated aramid, as shown in Table 3, the residual mass of modified AF-III with TiO_2_ in scCO_2_ was higher than those of the untreated fibers due to the better thermal stability of TiO_2_. 

### 3.5. Potential Mechanism of Modification

**Figure 13:**
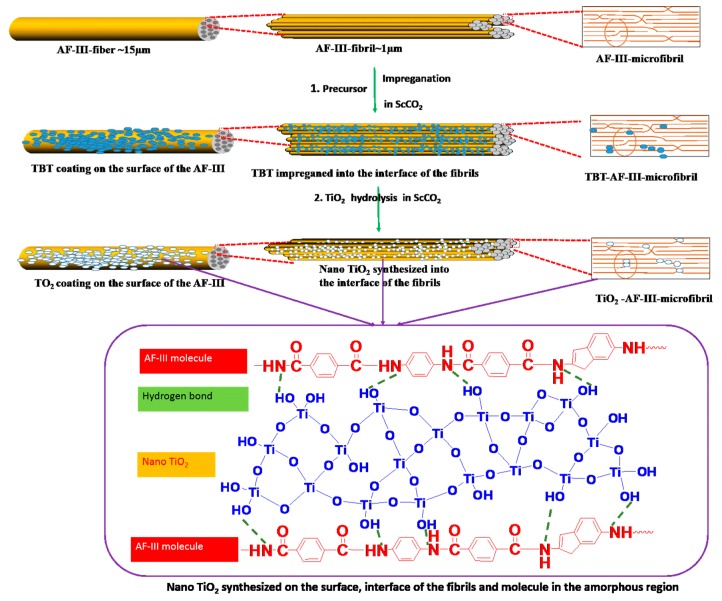
Potential mechanisms of modification in scCO_2_.

Based on the results and discussions above, a model of the modification process is shown in Figure 13. The AF-III fiber comprised fibrils with diameters ranging from 100 nm to 2 μm, which, in turn, are comprised of microfibrils [37]. The microfibrils are comprised of a large, perfectly oriented crystallite phase and an irregular amorphous phase. The amorphous phase has a less regular macromolecule with some chain end or defects, which exist between the crystallites or at fibril interfaces. Using scCO_2_ as a carrier, TBT as the TiO_2_ precursor could be dissolved and impregnated into the fibril interfaces, and deposited on the surface of the fiber when the CO_2_ was depressurized. In the second process step, TiO_2_ nanoparticles were synthesized via hydrolysis with ethanol. Plentiful hydroxyl groups on the surfaces of the fibers and the fibril/microfibril interfaces adsorb TiO^2+^, and then TiO_2_ was formed by hydrolysis condensation, subsequently growing into TiO_2_ particles. Meanwhile, the –CONH– groups of AF-III fiber would preferentially form rutile TiO_2_ [47]. In addition, hydrogen bonding exists between the hydroxyl groups of TiO_2_ and AF-III molecular chains; the chain end in the amorphous phase between the interfaces of the fibril will adhere to the fibrillar elements and provide a secondary interfibrillar force, which is useful for improving the mechanical properties.

## 4. Conclusions

In this study, a two-step process is proposed to synthesize nano-TiO_2_ on the surface of aramid fibers and in the interfacial space between the fibrils/microfibrils of AF-III in scCO_2_ to improve its UV-Vis resistance. The results of FTIR and XPS showed that the TiO_2_ particles were successfully prepared on the surface. In addition, the SEM results demonstrated that TiO_2_ was not only synthesized on the surface, but also in the interface between the microfibril/fibril. The effects of pressure during the preparation of TiO_2_ in scCO_2_ on the mechanical properties and morphology of aramid fibers were investigated. The results showed that, when treated at a pressure of 15 MPa, TiO_2_ is well synthesized and packed on the surface. It was found that the tensile strength and modulus of fibers treated in scCO_2_ were higher than those of untreated fibers. The tensile strength of TiO_2_-modified aramid fibers after photo-aging decreased less than that for untreated. This was because TiO_2_ could absorb UV and reduce the UV absorption of the aramid fiber itself. These findings showed that the scCO_2_ treatment process is a better method for improving the fiber’s properties, and could be a potential application of metal oxides to modify AFs and other high-performance fibers, which might lead to the development of exciting, new processes for engineers interested in bulletproof vests, firefighting gear components, ropes, and cables.

## Figures and Tables

**Table 1:** Chemical atomic compositions on the surface of fibers treated in scCO_2_.

Sample	Atomic Percent (%)			Atomic Ratio		
	C	N	O	Ti	O/C	Ti/C
untreated	76.85	5.71	17.44	0	0.2269	0
AF-III-scCO_2_-10MPa	76.38	4.49	18.52	0.61	0.2425	0.0080
AF-III-scCO_2_-12MPa	72.93	4.32	21.81	0.94	0.2991	0.0129
AF-III-scCO_2_-15MPa	67.98	4.13	25.23	2.66	0.3711	0.0391
AF-III-scCO_2_-20MPa	74.52	4.89	19.22	1.37	0.2579	0.0184

**Table 2:** Variation of TiO_2_ mass synthesized in scCO_2_.

Samples	Variation of TiO_2_ Mass			
	Pre-Reaction Mass (g)	Post-Reaction Mass (g)	TiO_2_ Content (mg)	TiO_2_ Added (wt %)
AF-III-scCO_2_-10MPa	0.2885	0.2913	2.8	0.97
AF-III-scCO_2_-12MPa	0.3154	0.3184	3.0	0.95
AF-III-scCO_2_-15MPa	0.3283	0.3361	7.8	2.38
AF-III-scCO_2_-20MPa	0.3019	0.3044	2.5	0.83

**Table 3:** Residual mass of the aramid fibers treated in scCO_2_ obtained from the TGA results.

Different Treatment Conditions	Residual Mass (%)
Untreated	48.11
AF-III-scCO_2_-10MPa	48.77
AF-III-scCO_2_-12MPa	52.70
AF-III-scCO_2_-15MPa	53.34
AF-III-scCO_2_-20MPa	51.18

## References

[1] Zhang S.H., He G.Q., Liang G.Z., Cui H., Zhang W., Wang B. (2010). Comparison of f-12 aramid fiber with domestic armid fiber iii on surface feature. Appl. Surf. Sci..

[2] Rodríguez-Uicab O., Avilés F., González-Chi P.I., Canché-Escamilla G., Duarte-Aranda S., Yazdani-Pedram M., Toro P., Gamboa F., Mazo M., Nistal A. (2016). Deposition of carbon nanotubes onto aramid fibers using as-received and chemically modified fibers. Appl. Surf. Sci..

[3] Ghosh L., Fadhilah M.H., Kinoshita H., Ohmae N. (2006). Synergistic effect of hyperthermal atomic oxygen beam and vacuum ultraviolet radiation exposures on the mechanical degradation of high-modulus aramid fibers. Polymer.

[4] Gu H. (2005). Ultraviolet treatment on high performance filaments. Mater. Des..

[5] Said M.A., Dingwall B., Gupta A., Seyam A.M., Mock G., Theyson T. (2006). Investigation of ultra violet (uv) resistance for high strength fibers. Adv. Space Res..

[6] Zhang H., Zhang J., Chen J., Hao X., Wang S., Feng X., Guo Y. (2006). Effects of solar uv irradiation on the tensile properties and structure of ppta fiber. Polym. Degrad. Stab..

[7] Jin J., Li G., Yang S., Jiang J. (2012). Effects of light stabilizer on the ultraviolet stability of poly-p-phenylenebenzobisoxazole (pbo) fibers. Iran. Polym. J..

[8] Lee Y.I., Jung M.H., Lee M.C. (2013). Improvement of photo-stability for p-aramid fibers by sio2/tio2sol-gel method. Text. Coloration Finish..

[9] Li S., Gu A., Xue J., Liang G., Yuan L. (2013). The influence of the short-term ultraviolet radiation on the structure and properties of poly(p-phenylene terephthalaramide) fibers. Appl. Surf. Sci..

[10] Pakdel E., Daoud W.A., Sun L., Wang X. (2014). Visible and uv functionality of tio2 ternary nanocomposites on cotton. Appl. Surf. Sci..

[11] Park S.M., Kwon I.J., Sim J.H., Lee J.H., Kim S.S., Lee M.C., Choi J.S. (2013). Improving the photo-stability of p-aramid fiber by tio2nanosol. Text. Coloration Finish..

[12] Cheng Z., Hong D., Dai Y., Jiang C., Meng C., Luo L., Liu X. (2018). Highly improved uv resistance and composite interfacial properties of aramid fiber via iron (iii) coordination. Appl. Surf. Sci..

[13] Kim Y. (2014). Ultraviolet protection finishes for textiles. Functional Finishes for Textiles: Improving Comfort, Performance and Protection.

[14] Mao N. (2014). High performance textiles for protective clothing. High Performance Textiles and Their Applications.

[15] Allen N.S., Edge M., Verran J., Stratton J., Maltby J., Bygott C. (2008). Photocatalytic titania based surfaces: Environmental benefits. Polym. Degrad. Stab..

[16] Yao J., Jin J., Lepore E., Pugno N.M., Bastiaansen C.W.M., Peijs T. (2016). Electrospinning of p-aramid fibers. Macromol. Mater. Eng..

[17] Xing Y., Ding X. (2007). Uv photo-stabilization of tetrabutyl titanate for aramid fibers via sol-gel surface modification. J. Appl. Polym. Sci..

[18] Deng H., Zhang H. (2015). In situ synthesis and hydrothermal crystallization of nanoanatase tio2-sio2 coating on aramid fabric (htisiaf) for uv protection. Microsc. Res. Tech..

[19] Walsh P.J., Hu X., Cunniff P., Lesser A.J. (2010). Environmental effects on poly-p-phenylenebenzobisoxazole fibers. I. Mechanisms of degradation. J. Appl. Polym. Sci..

[20] Walsh P.J., Hu X., Cunniff P., Lesser A.J. (2010). Environmental effects on poly-p-phenylenebenzobisoxazole fibers. II. Attempts at stabilization. J. Appl. Polym. Sci..

[21] Brown J.R., Browne N.M., Burchill P.J., Egglestone G.T. (1983). Photochemical ageing of kevlar 49. Text. Res. J..

[22] Arrieta C., David É., Dolez P., Vu-Khanh T. (2011). Hydrolytic and photochemical aging studies of a kevlar^®^-pbi blend. Polym. Degrad. Stab..

[23] Quye A. (2014). Factors influencing the stability of man-made fibers: A retrospective view for historical textiles. Polym. Degrad. Stab..

[24] Choi C., Yoon Y., Hong D., Brammer K.S., Noh K., Oh Y., Oh S., Talke F.E., Jin S. (2010). Strongly superhydrophobic silicon nanowires by supercritical co2 drying. Electron. Mater. Lett..

[25] Costa L.I., Storti G. (2017). Kinetic modeling of precipitation and dispersion polymerizations. Polymer Reaction Engineering of Dispersed Systems.

[26] Lee S.H., Park S., Kim M., Yoon D., Chanthad C., Cho M., Kim J., Park J.H., Lee Y. (2016). Supercritical carbon dioxide-assisted process for well-dispersed silicon/graphene composite as a li ion battery anode. Sci. Rep..

[27] Mumin M.A., Moula G., Charpentier P.A. (2015). Supercritical co2 synthesized tio2 nanowires covalently linked with core-shell cds-zns quantum dots: Enhanced photocatalysis and stability. RSC Adv..

[28] Ramsey E., Sun Q., Zhang Z., Zhang C., Gou W. (2009). Mini-review: Green sustainable processes using supercritical fluid carbon dioxide. J. Environ. Sci..

[29] Saarimaa V., Kaleva A., Nikkanen J.P., Heinonen S., Levänen E., Väisänen P., Markkula A., Juhanoja J. (2017). Supercritical carbon dioxide treatment of hot dip galvanized steel as a surface treatment before coating. Surf. Coat. Technol..

[30] Sanli D., Erkey C. (2015). Silylation from supercritical carbon dioxide: A powerful technique for modification of surfaces. J. Mater. Sci..

[31] Yeo S.D., Kiran E. (2005). Formation of polymer particles with supercritical fluids: A review. J. Supercrit. Fluids.

[32] Baseri S., Karimi M., Morshed M. (2012). Study of structural changes and mesomorphic transitions of oriented poly(ethylene therephthalate) fibers in supercritical CO_2_. Eur. Polym. J..

[33] Tao Y., Pescarmona P. (2018). Nanostructured oxides synthesised via scco2-assisted sol-gel methods and their application in catalysis. Catalysts.

[34] Yu Q., Wu P., Xu P., Li L., Liu T., Zhao L. (2008). Synthesis of cellulose/titanium dioxide hybrids in supercritical carbon dioxide. Green Chem..

[35] Zhang L., Kong H., Qiao M., Ding X., Yu M. (2019). Growing nano-sio2 on the surface of aramid fibers assisted by supercritical co2 to enhance the thermal stability, interfacial shear strength, and uv resistance. Polymers.

[36] Kong H., Teng C., Liu X., Zhou J., Zhong H., Zhang Y., Han K., Yu M. (2014). Simultaneously improving the tensile strength and modulus of aramid fiber by enhancing amorphous phase in supercritical carbon dioxide. RSC Adv..

[37] Kong H., Xu Q., Yu M. (2019). Microstructural changes of aramid fiber due to reaction with toluene 2,4-diisocyanate under tension in scCO_2_. Polymers.

[38] Ding X., Kong H., Qiao M., Hu Z., Yu M. (2019). Study on crystallization behaviors and properties of f-iii fibers during hot drawing in supercritical carbon dioxide. Polymers.

[39] Ahmed D., Zhong H., Kong H., Liu J., Ma Y., Yu M. (2014). The structural development and the thermal behaviours in the heat treated poly(p-phenylene terephthalamide) fibers. Fibers Polym..

[40] Wang B., Duan Y., Zhang J. (2016). Titanium dioxide nanoparticles-coated aramid fiber showing enhanced interfacial strength and uv resistance properties. Mater. Des..

[41] Byrappa K., Ohara S., Adschiri T. (2008). Nanoparticles synthesis using supercritical fluid technology—Towards biomedical applications. Adv. Drug Deliv. Rev..

[42] Kruse N., Chenakin S. (2011). Xps characterization of au/tio2 catalysts: Binding energy assessment and irradiation effects. Appl. Catal. A Gen..

[43] Wang D., Li L., Luo Q., An J., Li X., Yin R., Zhao M. (2014). Enhanced visible-light photocatalytic performances of ag3po4 surface-modified with small amounts of tio2 and ag. Appl. Surf. Sci..

[44] Xu Y., You H. (2014). Tio2 modified with ag nanoparticles synthesized via ultrasonic atomization-uv reduction and the use of kinetic models to determine the acetic acid photocatalytic degradation. Appl. Surf. Sci..

[45] Carlsson D.J., Gan L.H., Wiles D.M. (1978). Photodegradation of aramids. 11. Irradiation in air. J. Polym. Sci. Polym. Chem. Ed..

[46] Hamilton L.E., Sherwood P.M., Reagan B.M. (1993). X-ray photoelectron spectroscopy studies of photochemical changes in high-performance fibers. Appl. Spectrosc..

[47] Li Y., Cao L., Li L., Yang C. (2015). In situ growing directional spindle tio2 nanocrystals on cellulose fibers for enhanced pb(2+) adsorption from water. J. Hazard Mater..

